# Draft genome sequence data of the facultative, thermophilic, xylanolytic bacterium *Paenibacillus* sp. strain DA-C8

**DOI:** 10.1016/j.dib.2021.106784

**Published:** 2021-01-22

**Authors:** Chinda Chhe, Ayaka Uke, Sirilak Baramee, Umbhorn Ungkulpasvich, Chakrit Tachaapaikoon, Patthra Pason, Rattiya Waeonukul, Khanok Ratanakhanokchai, Akihiko Kosugi

**Affiliations:** a Biological Resources and Post-Harvest Division, Japan International Research Center for Agricultural Sciences (JIRCAS), 1-1 Ohwashi, Tsukuba, Ibaraki 305-8686, Japan; b Graduate School of Life and Environmental Sciences, University of Tsukuba, 1-1-1 Tennodai, Tsukuba, Ibaraki 305-8572, Japan; c Pilot Plant Development and Training Institute (PDTI), King Mongkut's University of Technology Thonburi (KMUTT), Bangkok 10150, Thailand; d Enzyme Technology Laboratory, School of Bioresources and Technology, King Mongkut's University of Technology Thonburi (KMUTT), Bangkok 10150, Thailand

**Keywords:** Draft genome, Facultative, Anaerobic bacteria, Thermophilic, Xylan degradation

## Abstract

Thermophilic, facultatively anaerobic, xylanolytic bacterial strain DA-C8 (=JCM34211 =DSM111723), newly isolated from compost, shows strong beechwood xylan degradation ability. Whole-genome sequencing of strain DA-C8 on the Ion GeneStudio S5 system yielded 69 contigs with a total size of 3,110,565 bp, 2,877 protein-coding sequences, and a G+C content of 52.3 mol%. Genome annotation revealed that strain DA-C8 possesses debranching enzymes, such as β-L-arabinofuranosidase and polygalacturonase, that are important for efficient degradation of xylan. As inferred from 16S rRNA sequences and average nucleotide identity values, the closest relatives of strain DA-C8 are *Paenibacillus cisolokensis* and *P. chitinolyticus*. The genomic data have been deposited at the National Center for Biotechnology Information (NCBI) under accession number BMAQ00000000.

## Specifications Table

**Table:** 

Subject	Microbiology
Specific subject area	Bacteriology, Genomics
Type of data	Figure, Supplementary table
How data were acquired	Whole-genome sequencing using the Ion GeneStudio S5 System
Data format	Raw, Analyzed
Parameters for data collection	Genomic DNA was extracted from a pure culture of strain DA-C8 (DSM 111723). The genome of strain DA-C8 was sequenced on the Ion GeneStudio S5 system, *de novo* assembled using CLC Genomics Workbench 20.0.1, and annotated using the DDBJ Fast Annotation and Submission Tool (DFAST).
Description of data collection	Genomic DNA was extracted from strain DA-C8. A sequencing library with an insert size of 300–400 bp was prepared using an Ion Xpress Plus Fragment Library kit (Thermo Fisher Scientific, Waltham, MA, USA). Approximately 200–300-bp fragments were size-selected by electrophoresis on E-Gel SizeSelect II agarose gels (Invitrogen, Thermo Fisher Scientific) before library preparation. The genomic library of strain DA-C8 was subjected to whole-genome sequencing, assembly, and annotation.
Data source location	Japan International Research Center for Agricultural Sciences (JIRCAS), Tsukuba, Ibaraki, Japan
Data accessibility	The draft genome sequence has been deposited at DDBJ/ENA/GenBank under accession number BMAQ00000000. The direct URL to the data is https://www.ncbi.nlm.nih.gov/nuccore/BMAQ00000000.1. BioProject and BioSample IDs in GenBank are PRJDB10171 (https://www.ncbi.nlm.nih.gov/bioproject/PRJDB10171) and SAMD00235398 (https://www.ncbi.nlm.nih.gov/biosample/SAMD00235398).
	All additional data analysis files and supplementary tables can be accessed at Mendeley Data (http://dx.doi.org/10.17632/5gzxp24s4z.2).

## Value of the Data

• The genome data from newly isolated strain DA-C8 contribute to understanding of mechanisms of efficient degradation of lignocellulosic biomass, including xylan, by xylanolytic bacteria.
• Comparison of the genome data of strain DA-C8 with data of other xylanolytic bacteria can yield information useful for enhancing the efficiency of xylanolytic enzymes.
• The genome data of strain DA-C8 can aid taxonomic delineation of new independent genera and *Paenibacillus*.

## 1 Data Description

Efficient hydrolysis of lignocellulosic biomass not only requires the participation of β-1,4-glycosidic chain-cleaving enzymes, such as endo-β-1,4-glucanase, cellobiohydrolases, and β-glucosidase, but also the cooperation of numerous hemicellulosic enzymes (e.g., xylanolytic enzymes) and side chain-cleaving enzymes (e.g., α-L-arabinofuranosidase) [1]. Cellulolytic and xylanolytic enzymes, in particular, have various potential industrial applications in a wide variety of areas, such as food engineering and the production of supplements, animal feed, bio-ethanol, and pulp [2], [3], [4]. The laundry and dish detergent industry is one of the primary consumers of industrial enzymes [2], [3], [5]. Among xylanolytic enzymes, *Paenibacillus* strains produce a variety of enzymes, including amylases, cellulases, xylanases, other hemicellulases, and lipases, with potential applications to the industrial manufacturing of detergents, food, paper, and biofuels [5]. Enzymes of *Paenibacillus* strains are highly active under industrially-relevant conditions, and *Paenibacillus* strains can be produced at a lower cost than available alternatives by high density culture [5].

The screening, identification, and characterization of the functional properties of strongly xylanolytic bacteria are of crucial importance for the construction of applicable bioprocesses. To obtain a bacterium exhibiting efficient xylan-degradation ability under anaerobic and thermophilic conditions, we newly isolated strain DA-C8, assigned to the genus *Paenibacillus*, as a pure culture from compost. This strain was deposited at the RIKEN BioResource Research Center as JCM 34211 and at the German Collection of Microorganisms and Cell Cultures GmbH (DSMZ) as DSM111723. Strain DA-C8 possesses strong xylan-degradation ability under thermophilic anaerobic conditions. We compared the xylan-degradation abilities of DA-C8 and *P. curdlanolyticus* B-6, which is highly xylanolytic because of the production of an extracellular multienzyme complex [6], using beechwood xylan (1% w/v). When we incubated DA-C8 and B-6 for 6 days at 55 °C under anaerobic conditions in previously reported BMN basal medium [7] or at 37 °C under aerobic conditions in Berg′s mineral salt medium [6], respectively, complete degradation of beechwood xylan was achieved earlier with strain DA-C8. Strain DA-C8 can thus degrade beechwood xylan more efficiently than can xylanolytic *P. curdlanolyticus* B-6.

We sequenced the whole genome of strain DA-C8 to obtain information on the effective, strong xylan-degradation system of this bacterium. DNA sequencing performed using the Ion GeneStudio S5 system [8] generated 11,760,377 reads. *De novo* genome assembly using CLC Genomics Workbench 20.0.1 (CLC Bio, Qiagen, Valencia, CA) yielded 69 contigs with an N50 of 108,510 bp and a mean contig length of 45,081 bp. The genome of strain DA-C8 comprised 3,110,565 bp and had a G+C content of 52.3 mol%. Genome annotation was carried out via the DDBJ Fast Annotation and Submission Tool (DFAST). Strain DA-C8 had 2,877 protein-coding sequences, 2 rRNA genes, 55 tRNA genes, and 5 CRISPR genes. We obtained a 650-fold genome coverage depth.

**Fig. 1:**
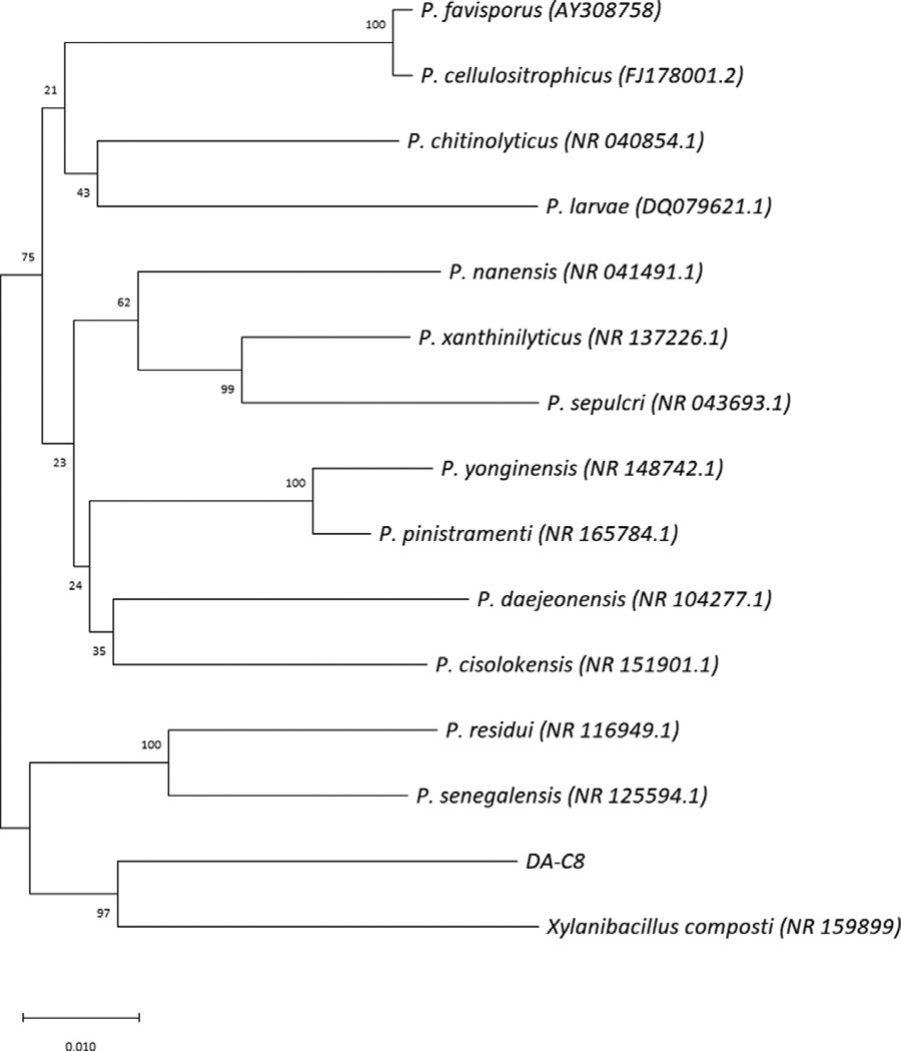
Neighbor-joining tree depicting the relationships of 13 *Paenibacillus* isolates, *Xylanibacillus composti*, and strain DA-C8 based on 16S rRNA sequences. Numbers at nodes are bootstrap support percentages based on 1,000 replicates. The bar represents 0.01 substitutions per nucleotide position.

**Fig. 2:**
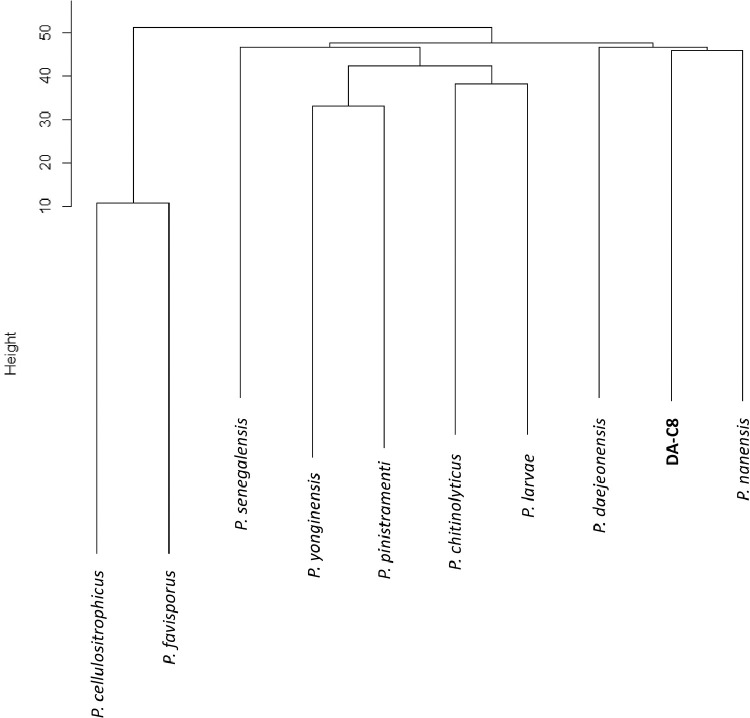
Dendrogram of average nucleotide identity (ANI) values. ANI values between different *Paenibacillus* strains were calculated and used to construct a dendrogram based on the unweighted pair group method with arithmetic means. The following 10 strains were used: DA-C8 (BMAQ00000000.1), *P. cellulositrophicus* (CP045295), *P. chitinolyticus* (NZ_CP026520); *P. daejeonensis* (ARKE00000000.1), *P. favisporus* (WIBG00000000.1), *P. larvae* (NZ_CP019687), *P. nanensis* (QXQA00000000.1), *P. pinistramenti* (VAWG00000000.1), *P. senegalensis* (CAES00000000.1), and *P. yonginensis* (CP014167).

Phylogenetic analysis based on 16S rRNA sequences (Fig. 1) revealed a close similarity between strain DA-C8 and the following strains: *P. cisolokensis* UICC B-42 (93.7% identity; accession no. NR_151901)*, Xylanibacillus composti* K-13 (92.8% identity; NR_159899)*, P. pinistramenti* ASL46 (92.7% identity; LC102482), *P. senegalensis* JC66 (92.6% identity; NR_125594), *P. yonginensis* DCY84 (92.5% identity; NR_148742), *P. chitinolyticus* (92.4% identity; NR_040854)*, P. favisporus* Y7 (92.4% identity; AY308758)*, P. residui* MC-246P (92.2% identity; NR_116949) (Supplementary table 1) [9]. The closest relatives of strain DA-C8 based on average nucleotide identity (ANI) values with nine other *Paenibacillus* strains were *P. chitinolyticus* KCMM41400 (68.7%), followed by *P. cellulositrophicus* KACC16577 (68.1%), *P. yonginensis* DCY 84 (68.1%), and *P. larvae* (67.7%) (Fig. 2, and supplementary tables 2 and 3) [9]. ANIs between DA-C8 and all additional strains included in the analysis were < 70% (Fig. 2 and supplementary table 3) [9].

The genome annotation confirmed the presence of the following predicted essential enzymes having xylan and lignocellulosic biomass degradation abilities in strain DA-C8: endo-1,4-β-xylanase (EC 3.2.1.8), acetylxylan esterase (EC 3.1.1.72), α-L-arabinofuranosidase (EC 3.2.1.55), β-L-arabinofuranosidase (EC 3.2.1.185), α-xylosidase (EC 3.2.1.177), β-xylosidase (EC 3.2.1.37), β-glucosidase (EC 3.2.1.21), endo-1,4-β-glucanase (EC 3.2.1.4), α-glucoamylase (EC 3.2.1.3), polygalacturonase (EC 3.2.1.15), and α-glucuronosidase (EC 3.2.1.131). Of particular interest, the detected debranching enzymes, such as β-L-arabinofuranosidase and polygalacturonase, are not present in the genome sequence of *P. curdlanolyticus* B-6 [8]. The contigs and annotated data of strain DA-C8 can be accessed at Mendeley Data [9].

## 2 Experimental Design, Materials and Methods

### 2.1 Bacterial strain isolation and deposition into collections

Strain DA-C8 was isolated from compost as described previously. Modified BMN medium [7], which consisted of 2.9 g/L K_2_HPO_4_, 4.2 g/L urea, 2.0 g/L yeast extract, 1.0 g/L Na_2_CO_3_, 0.01 g/L CaCl_2_·2H_2_O, 0.5 g/L cysteine-HCl, and 0.0005 g/L resazurin in water and 200 µL aqueous mineral solution (25.0 g/L MgCl_2_·6H_2_O, 37.5 g/L CaCl_2_·2H_2_O, and 0.312 g/L FeSO_4_·7H_2_O) supplemented with 1% (w/v) beechwood xylan as the sole carbon source, was used as the basal medium. All chemicals used for the basal medium were purchased from Fujifilm Wako Pure Chemicals, Osaka, Japan. The basal medium was aerated with high-purity nitrogen gas before autoclaving. Strain DA-C8 (=JCM34211=DSM111723) was deposited in the open culture collection of the RIKEN Bioresource Research Center (JCM) and the Leibniz Institute German Collection of Microorganisms and Cell Cultures (DSMZ). The culture of DA-C8 was centrifuged, and the pellet was used for DNA extraction. *P. curdlanolyticus* B-6 was cultivated on Berg's mineral salt medium at 37 °C under aerobic shaking conditions [6].

### 2.2 Genomic DNA purification and sequencing

After cultivation of cells for 4 days under anaerobic conditions at 55 °C with xylose as the carbon source, genomic DNA was extracted by the phenol/chloroform method [8] and purified. DNA fragmentation and library preparation were carried out using an Ion Xpress Plus Fragment Library kit (catalog no. #4471269, Thermo Fisher Scientific, Waltham, MA, USA) according to the manufacturer's instructions. Before library preparation, fragments approximately 200 to 300 bp in size were selected by electrophoresis on Invitrogen E-Gel SizeSelect II agarose gels (catalog no. #G661012, Thermo Fisher Scientific). Genomic DNA sequences of strain DA-C8 were obtained using the Ion GeneStudio S5 system and then processed [8].

### 2.3 Phylogenetic analysis

Sequences obtained by BLAST searching against the GenBank database were manually aligned with the 16S rRNA sequence of strain DA-C8 using CLUSTAL_W [10]. A phylogenetic tree was generated by the neighbor-joining method based on the Tamura-3 parameter model [11] in MEGA X v10.1 [12].

### 2.4 Genome assembly, annotation, and analysis

Trimming of low-quality raw sequences and *de novo* genome assembly were performed in CLC Genomics Workbench v20.0.1. The genome assembly was annotated using DDBJ DFAST (https://dfast.nig.ac.jp/). Protein families of predicted essential xylan degradation enzymes were identified with CAZymes (http://www.cazy.org/).

### 2.5 Genomic ANIs

Calculation of pairwise ANI values of whole-genome sequences of strain DA-C8 and nine *Paenibacillus* strains was conducted in GENETYX NGS v4.1.1. The matrix generated from the calculated ANI values was converted into a genetic dendrogram using algorithms described previously [8].

## Ethics Statement

This research and analysis did not involve the use of human subjects or animal experiments.

## Declaration of Competing Interest

The authors declare that they have no known competing financial interests or personal relationships which have or could be perceived to have influenced the work reported in this article.

## Appendix Supplementary materials

Image, image 1

Image, application 2

Image, application 3

Image, application 4

Image, application 5

## Footnotes

Supplementary material associated with this article can be found in the online version at doi:10.1016/j.dib.2021.106784.
