# Supplementary figures and images for: Draft genome sequence data of the facultative, thermophilic, xylanolytic bacterium Paenibacillus sp. strain DA-C8
Source: Data Brief. 2021 Jan 22;35:106784. doi: 10.1016/j.dib.2021.106784 (PMC7859314; doi:10.1016/j.dib.2021.106784)

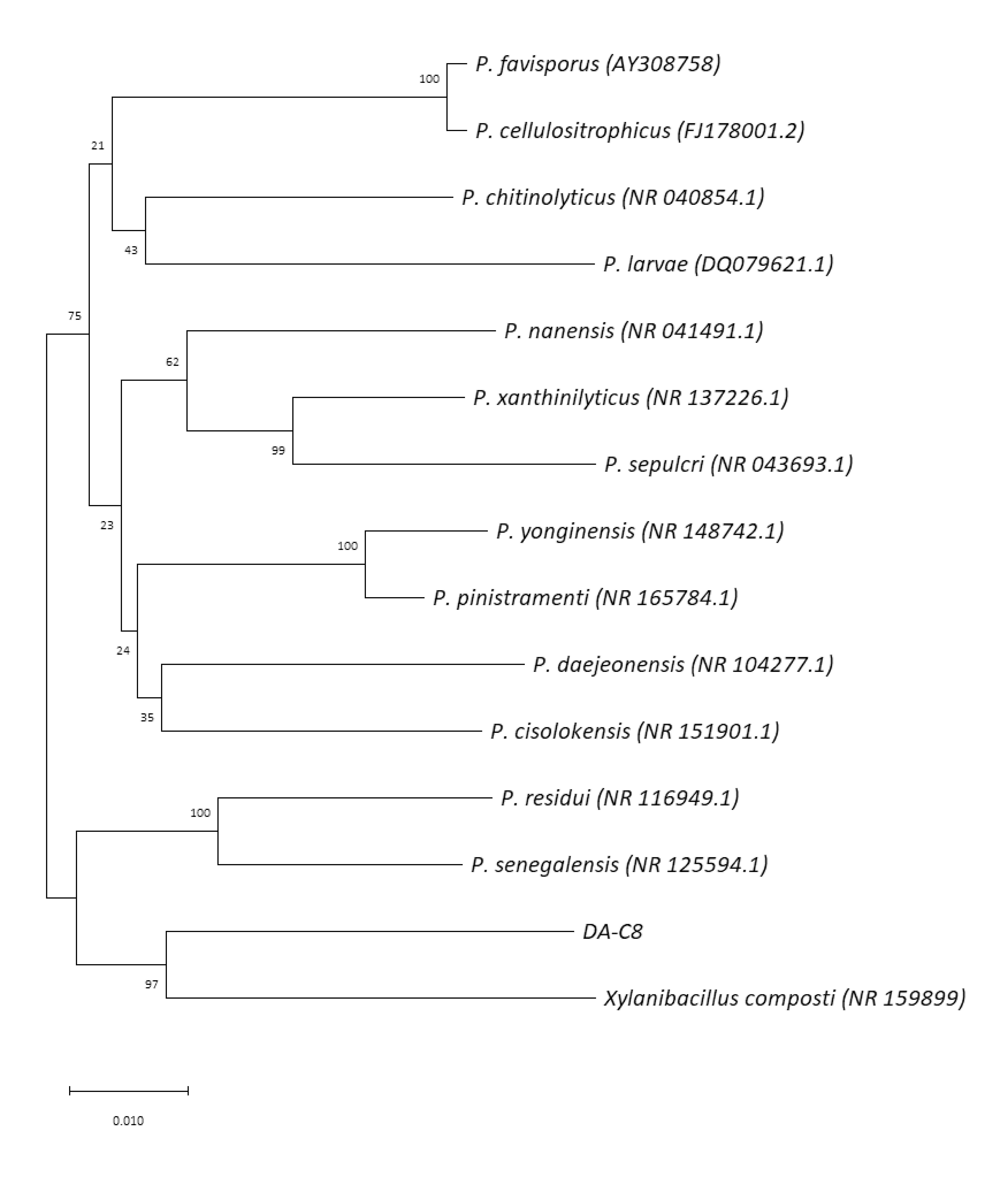

Supplement: Supplementary file 1 [file mmc1.jpg]

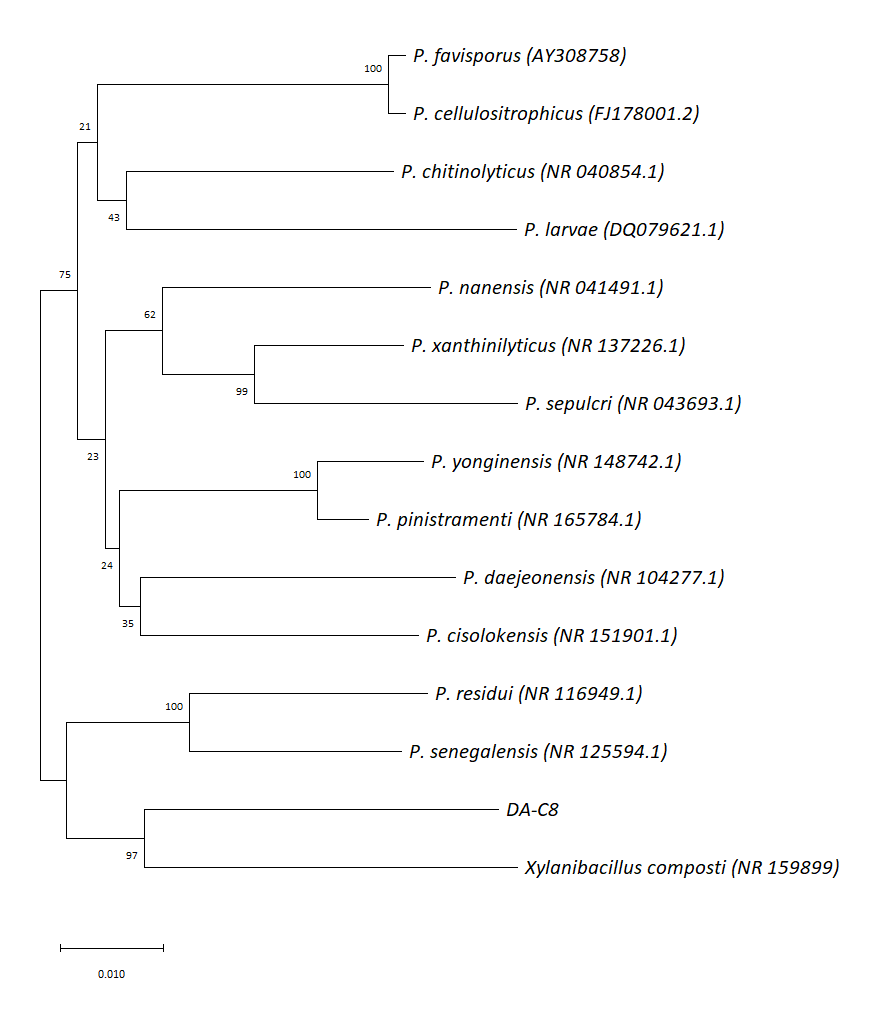

Supplement: Supplementary file 3 [file mmc3.zip › mmc3.tif]

## Slide 1
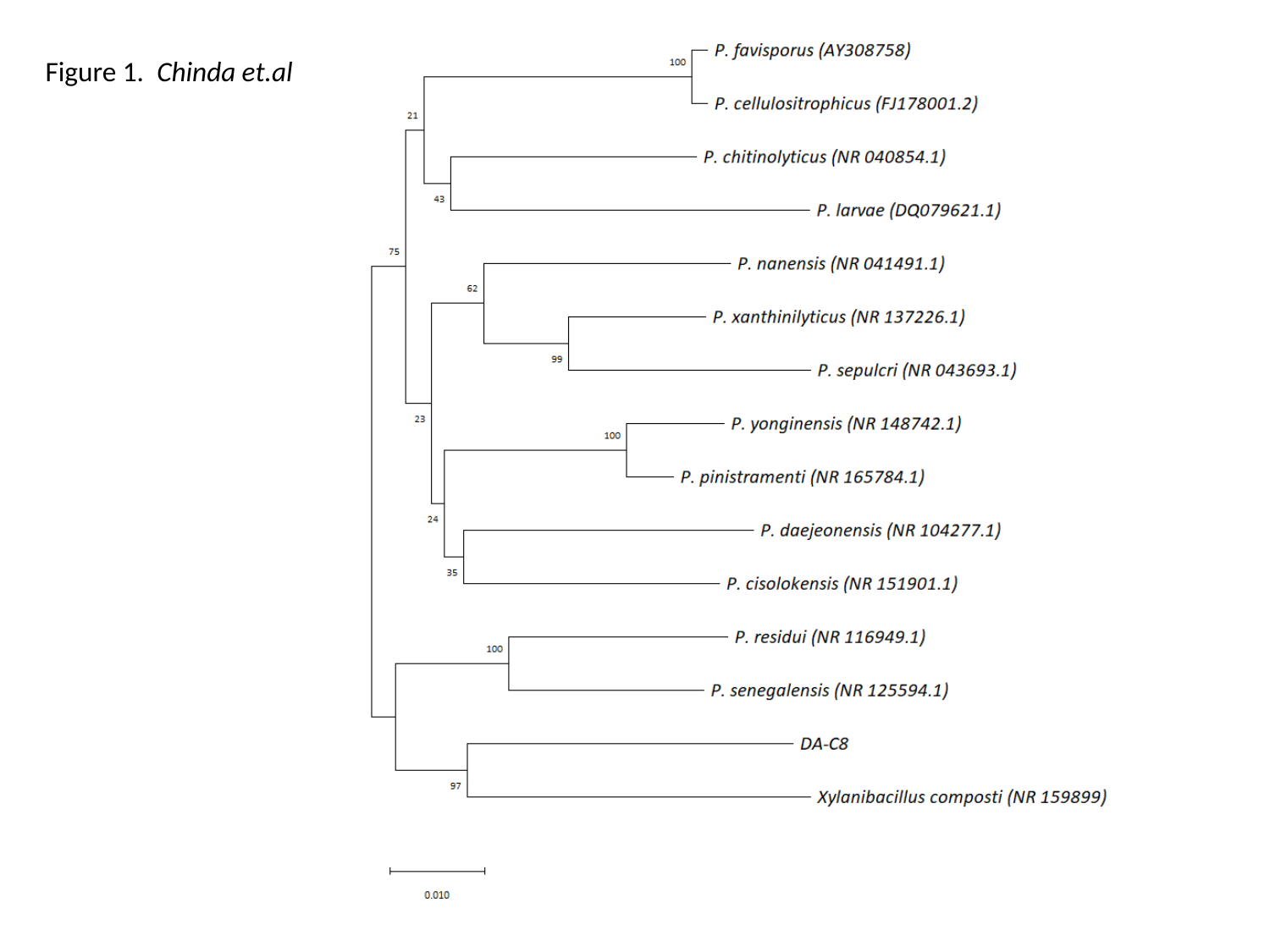

Figure 1. Chinda et.al

## Slide 2
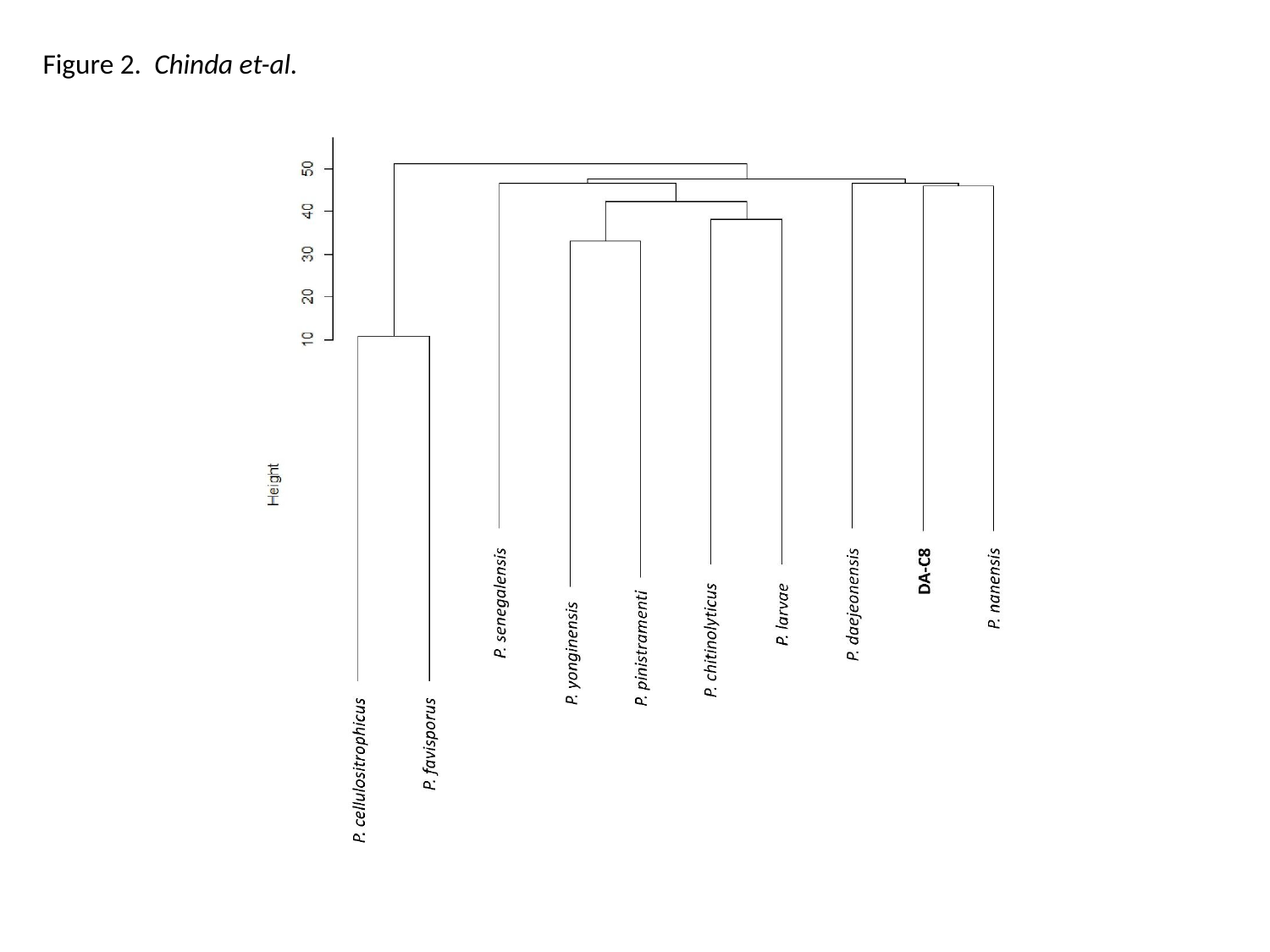

Figure 2. Chinda et-al.

Supplement: Supplementary file 4 [file mmc4.pptx]
